# Dietary salt regulates uroguanylin expression and signaling activity in the kidney, but not in the intestine

**DOI:** 10.14814/phy2.12782

**Published:** 2016-05-15

**Authors:** Robert C. Fellner, Nicholas G. Moss, Michael F. Goy

**Affiliations:** ^1^ Department of Cell Biology and PhysiologyUniversity of North Carolina at Chapel HillChapel HillNorth Carolina

**Keywords:** Dietary salt, entero‐renal endocrine axis, natriuretic peptide, sodium homeostasis

## Abstract

The peptide uroguanylin (Ugn) is expressed at significant levels only in intestine and kidney, and is stored in both tissues primarily (perhaps exclusively) as intact prouroguanylin (proUgn). Intravascular infusion of either Ugn or proUgn evokes well‐characterized natriuretic responses in rodents. Furthermore, Ugn knockout mice display hypertension and salt handling deficits, indicating that the Na^+^ excretory mechanisms triggered when the peptides are infused into anesthetized animals are likely to operate under normal physiological conditions, and contribute to electrolyte homeostasis in conscious animals. Here, we provide strong corroborative evidence for this hypothesis, by demonstrating that U_U__gn_V (the rate of urinary Ugn excretion) approximately doubled in conscious, unrestrained rats consuming a high‐salt diet, and decreased by ~15% after salt restriction. These changes in U_U__gn_V were not associated with altered plasma proUgn levels (shown here to be an accurate index of intestinal proUgn secretion). Furthermore, enteric Ugn mRNA levels were unaffected by salt intake, whereas renal Ugn mRNA levels increased sharply during periods of increased dietary salt consumption. Together, these data suggest that diet‐evoked Ugn signals originate within the kidney, rather than the intestine, thus strengthening a growing body of evidence against a widely cited hypothesis that Ugn serves as the mediator of an entero‐renal natriuretic signaling axis, while underscoring a likely intrarenal natriuretic role for the peptide. The data further suggest that intrarenal Ugn signaling is preferentially engaged when salt intake is elevated, and plays only a minor role when salt intake is restricted.

## Introduction

The classical mechanisms that regulate electrolyte homeostasis require several days to reestablish equilibrium after a large shift in Na^+^ intake (Simpson 1988). However, like many animals (including humans), salt intake in rats typically occurs over relatively brief periods at widely spaced intervals (Stoynev et al. 1982), a feeding pattern that is likely to cause short‐term imbalances in electrolyte homeostasis. It has been proposed that this situation is avoided by rapid buffering mechanisms that are activated by the arrival of Na^+^ in the intestinal lumen (Lennane et al. 1975; Carey 1978; Forte 2003).

The natriuretic peptide uroguanylin (Ugn) could mediate such a buffering mechanism through the operation of a postulated entero‐renal signaling axis (Forte et al. 1996, 2000; Krause et al. 1997; Potthast et al. 2001; Forte 2003; Elitsur et al. 2006; Qian et al. 2008; Lima and Fonteles 2014). According to this hypothesis, ingestion of Na^+^ causes endocrine cells in the intestinal epithelium to secrete proUgn, which is the predominant form of the peptide stored in tissues and circulating in the plasma (Moss et al. 2008). Upon delivery to the renal tubules and the lumen of the gut, the secreted proUgn is processed to Ugn (Hamra et al. 1996; Qian et al. 2008), leading to increased Na^+^ excretion in the urine (Greenberg et al. 1997; Fonteles et al. 1998; Nakazato et al. 1998; Carrithers et al. 2004; Elitsur et al. 2006; Lessa et al. 2012) and decreased Na^+^ absorption from the intestine (Joo et al. 1998; Donowitz et al. 2005; Toriano et al. 2011).

This proposed entero‐renal signaling mechanism requires that plasma proUgn levels rise when a meal is ingested, and fall during a fast. Indeed, transient postprandial increases in circulating proUgn have been described in mice and humans in response to a complex meal (Valentino et al. 2011). However, a recent study in humans failed to link this response with sodium intake, reporting instead that plasma proUgn levels actually decreased briefly after an acute oral salt challenge (Preston et al. 2012). This suggests that acute enteric proUgn secretion may be triggered by some component of food other than Na^+^, and therefore, does not provide a means to induce a rapid natriuretic response following dietary salt intake.

An alternative possibility is that proUgn and/or Ugn carry out a longer term integrative role in Na^+^ homeostasis, responding relatively slowly to chronic changes in salt consumption. Indeed, this idea fits well with the hypertensive phenotype of the Ugn knockout mouse (Lorenz et al. 2003), in which the chronically elevated blood pressure could represent a compensatory response to the loss of a chronic natriuretic activity. The experiments described here were designed to critically evaluate the hypothesis that long‐term changes in oral Na^+^ intake evoke long‐term physiological adjustments in the Ugn signaling pathway. Rats were sequentially exposed to diets with normal, restricted, and elevated Na^+^. We measured the effects on Ugn mRNA in intestine and kidney, and on proUgn and Ugn in plasma and urine. Measurements were made at defined times during the dark–light cycle to control for circadian variability. Our results show that Ugn production is indeed responsive to alterations in Na^+^ intake but that this is very likely restricted to renal Ugn production. Enteric proUgn expression and secretion appear to be insensitive to salt intake.

## Methods

### Animals

Experiments were performed on male Sprague‐Dawley rats (350 ± 28 g, Harlan laboratories, Dublin VA), and male C57BL6 mice, (wild‐type and Ugn knockouts backcrossed >10 generations into the BL6 background, generously provided by Dr. Mitchell Cohen). Animals were maintained on a fixed light/dark cycle (8 am/8 pm) in an AALAC‐approved facility with continuous veterinary care. Tap water and commercial rodent diets were consumed ad libitum. Experimental protocols and procedures were approved by the UNC IACUC, and are in accordance with the NIH Guide for the Care and Use of Laboratory Animals.

### Peptides

Rat guanylin (Gn) and Ugn were purchased from Anaspec Inc., San Jose CA and Peptides International Inc., Boston, MA. N‐tyrosyl rat Ugn (Y‐Ugn, Bachem Inc. Torrance, CA) was iodinated as previously described (Moss et al. 2010). Recombinant rat proUgn was kindly provided by Ironwood Pharmaceuticals (Cambridge, MA; Moss et al. 2009).

### Metabolic studies

Animals were housed individually in metabolic cages (Techniplast, Milan, Italy) and allowed 2 days for acclimation before sample collection. Animals were initially provided with a defined diet containing normal levels of NaCl (NS) (0.26% Na^+^), followed consecutively by low‐salt chow (LS) (0.02% Na^+^), high‐salt chow (HS) (3.1% Na^+^), and a return to NS chow (all diets purchased from Harlan Teklad, Madison, WI). Food and water consumed, and urine and feces excreted, were measured gravimetrically every 6 h (Table 1). Urine was collected under mineral oil. Na^+^ and K^+^ concentrations were measured by flame photometry (Model 943 Flame Photometer, Instrumentation Laboratory Co., Lexington, MA). Arterial plasma was sampled from some animals via an indwelling carotid cannula for plasma proUgn measurements. Additional animals were killed at various times to obtain plasma samples, along with contemporaneous tissue samples.

**Table 1:** Metabolic data

	Day																		
	1	2	3	4	5	6	7	8	9	10	11	12	13	14	15	16	17	18	19
	0.26% Na^+^ Chow				0.02% Na^+^ Chow					3.1% Na^+^ Chow					0.26% Na^+^				
Food intake (g)	25.79 ± 1.16	25.40 ± 0.83	27.68 ± 1.24	26.33 ± 1.12	26.99 ± 1.21	27.53 ± 1.55	27.05 ± 1.61	25.57 ± 1.70	24.61 ± 0.92	20.96 ± 2.18	22.65 ± 1.8	23.89 ± 1.70	24.01 ± 2.01	23.60 ± 1.24	28.42 ± 0.66	30.13 ± 1.29	30.67 ± 0.99	28.52 ± 1.17	28.95 ± 0.93
Water intake (mL)	31.46 ± 1.49	35.02 ± 1.50	36.59 ± 1.58	33.73 ± 1.23	27.04 ± 1.49	30.33 ± 1.30	32.65 ± 1.84	33.68 ± 2.53	35.57 ± 3.02	76.53 ± 5.31	85.11 ± 5.73	94.73 ± 4.65	99.68 ± 6.94	91.45 ± 5.01	37.93 ± 2.96	35.16 ± 3.72	37.92 ± 1.06	34.38 ± 0.29	37.74 ± 2.07
Na+ intake (mEq)	2.58 ± 0.13	2.87 ± 0.09	2.98 ± 0.14	3.45 ± 0.23	0.23 ± 0.01	0.24 ± 0.01	0.24 ± 0.01	0.23 ± 0.01	0.22 ± 0.01	28.91 ± 2.37	31.39 ± 1.87	32.95 ± 1.86	33.04 ± 2.16	31.81 ± 1.68	3.21 ± 0.07	3.41 ± 0.15	3.47 ± 0.11	3.22 ± 0.13	3.27 ± 0.10
UNa+ excretion (mEq)	1.50 ± 0.08	1.76 ± 0.04	1.84 ± 0.23	1.99 ± 0.23	0.54 ± 0.07	0.13 ± 0.01	0.11 ± 0.01	0.11 ± 0.01	0.11 ± 0.01	16.88 ± 0.87	26.94 ± 2.31	30.57 ± 1.44	31.51 ± 2.62	27.89 ± 1.44	3.67 ± 0.83	1.46 ± 0.53	1.68 ± 0.69	2.63 ± 0.16	2.55 ± 0.15
Urine flow (µL/min)	8.06 ± 0.42	8.67 ± 0.31	10.9 ± 1.97	9.75 ± 1.1	8.51 ± 0.92	9.51 ± 1.03	9.93 ± 0.68	10.48 ± 1.17	10.73 ± 1.27	29.83 ± 3.29	42.77 ± 4.9	48.5 ± 5.14	49.61 ± 6.94	45.15 ± 5.03	13.68 ± 1.39	11.12 ± 0.11	10.66 ± 0.51	10.22 ± 0.64	10.38 ± 0.63
Sample size	3	3	7	10	10	10	10	10	10	10	10	10	10	10	7	3	3	3	3

The table shows the effects of diet changes on the 24‐h consumption and excretion of food, water, Na^+^, and fluid for animals included in the diet study.

### Tissue ligation studies

Rats were anesthetized (sodium pentobarbital, 60 mg/kg, ip) and prepared for clearance experiments as previously described (Moss et al. 2010). They received an initial infusion of isotonic saline (6% of body weight), followed by an infusion of 10% mannitol in 0.9% saline at 100 *μ*L/min containing FITC‐labeled inulin (400 *μ*g/mL, Sigma Chemical Co. St Louis, MO). For renal ligations, 5.0 silk thread ligatures were placed loosely around right and left renal arteries, and tightened after baseline data had been collected. For intestinal ligations, the bowels were exteriorized and wrapped in saline‐soaked gauze. Ligatures were consecutively placed around the superior mesenteric artery, celiac artery, and portal vein, and tightened in order, allowing pressure in the downstream vasculature to drop before tightening the next ligature. Blood was sampled every 40 min from the femoral artery.

### Processing of plasma and tissues

Blood was collected into heparinized tubes (Sigma Chemical Company, St. Louis, MO). Plasma was isolated by centrifugation (16,000 × *g* for 5 min), and fractionated on a Superdex size exclusion column (GE Healthcare, Life Sciences, Piscataway, NJ) prior to western blot analysis of proUgn levels. Proximal small intestine (10 cm, starting 2 cm beyond the pyloric sphincter) was cut longitudinally, and rinsed with saline. Each kidney was flushed intravascularly with saline to clear it of plasma and ultrafiltrate. Tissues were frozen on dry ice, and either minced and stored at 4°C in RNAlater (Qiagen Inc., Valencia, CA) for qRT‐PCR, or frozen on dry ice, homogenized, centrifuged, and the supernatant fraction was mixed with SDS sample buffer and boiled for Western blot analysis.

### qRT‐PCR

Random hexamer‐primed cDNA was prepared from isolated RNA, using the SuperScript^®^ III First‐Strand Synthesis System (Invitrogen, Carlsbad, CA), according to the supplier's protocol. Amplifications were performed in duplicate for 40 thermal cycles (15 sec at 94°C and 1 min at 60°C, primer pairs and probes are shown in Table 2), as described by Kim et al. (2002). The fractional cycle at which each sample crossed a fluorescence threshold, CT, was determined using the manufacturer's software.

**Table 2:** Primers and probes used for qRT‐PCR

Primer set	Forward primer	Reverse primer	Probe
A (proUgn)	c(a/c)cagggtgtctacatcaag	tctc(c/t)tccaactcattcagc	ftggcttccaagtccagctg/agaatcq
B (β‐actin)	tgcctgacggtcaggtca	caggaaggaaggctggaag	fcactatcggcaatgagcggttccgq

The parenthesized portions of the primer sequences indicate positions where two nucleotides were incorporated in a 1:1 ratio, to allow use with both rats and mice; f and q represent the fluorophore (FAM) and quencher (Tamra) added to the probes, as required to generate the Taqman signal.

### ProUgn assay

ProUgn concentrations in plasma and tissues were measured by quantitative western blotting, as described previously (Moss et al. 2008). Samples were loaded on 4–12% polyacrylamide gels (Invitrogen Corp., Carlsbad, CA), and a standard curve was constructed with known amounts of recombinant rat proUgn (Ironwood Pharmaceuticals, Cambridge, MA). Gels were blotted to nitrocellulose and probed with primary antibody 6910 (Moss et al. 2008; Qian et al. 2008) and an IR dye‐coupled secondary antibody. Imaging and quantification were performed with an Odyssey System (LI‐COR Biosciences, Lincoln, NE). Values were corrected for recovery, as determined in parallel control assays with known amounts of recombinant rat proUgn.

**Figure 1:**
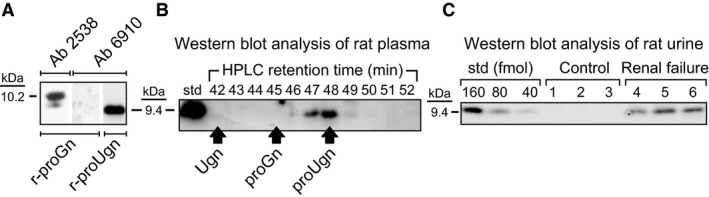
Characterization of the antibody used for the proUgn western blot assay. (A) Experiments with recombinant propeptides demonstrate the selectivity of anti‐proUgn antibody 6910. The two left lanes were loaded with identical samples of purified recombinant proGn that were run side‐by‐side on the same gel, then separated for incubation with different primary antibodies, as indicated. Anti‐proGn antibody 2538 recognizes proGn (the minor immunopositive band of lower molecular weight presumably represents small amounts of a proGn degradation product), but antibody 6910 does not. In contrast, antibody 6910 selectively recognizes purified recombinant proUgn (right lane). (B) Rat plasma (50 *μ*L) was fractionated by C‐18 reverse‐phase HPLC, and then 500 *μ*L of each 1 mL fraction was dried, resuspended in electrophoresis sample buffer, and analyzed by immunoblotting. Antibody 6910 should not recognize Ugn or proGn, and the blot confirms a complete lack of crossreactivity at retention times where Ugn or proGn would elute from the column. Here, and in all other relevant figures, retention times for recombinant proUgn, proGn, and Ugn standards were established in parallel column runs, and are indicated by the arrows. The left‐most lane of the gel was loaded with a rat proUgn standard. (C) Western blot analysis of urine samples obtained from three normal rats (control) and three animals previously subjected to surgical ablation of five‐sixths of their renal mass (renal failure). Each urine sample (100 *μ*L) was prefractionated on a Superdex column, as described in the Methods, and then fractions 31–34 (where proUgn elutes from this column) were pooled, dried, resuspended in electrophoresis sample buffer, and half of this final sample was analyzed by immunoblotting. The left‐hand lanes of the gel were loaded with a dilution series of recombinant rat proUgn, as a standard curve.

The properties and selectivity of the antibody used in this assay (Ab 6910) are shown in Figure 1. In the rat, proguanylin (proGn) is the only polypeptide that shares sequence homology with proUgn. However, the antigen used to raise antibody 6910 was derived from a region of proUgn that is poorly conserved in proGn. Therefore, as would be expected, 6910 recognizes recombinant proUgn in our assay, but does not recognize recombinant proGn (Fig. 1A). Note further that intact proUgn is readily detectable in HPLC‐fractionated plasma (Fig. 1B), but consistent with previous studies (Kinoshita et al. 1997; Nakazato et al. 1998; Kikuchi et al. 2005), is detectable in rat urine only after experimentally induced kidney failure [here generated by surgical ablation of five‐sixths of the animal's renal mass (Morrison 1962)] (Fig. 1C)**.**

### Quantitative Ugn binding assay

Urine samples were first dialyzed against deionized H_2_O, using SpectraPor^®^ 500‐M.W. cutoff dialysis membrane (Spectrum Laboratories Inc., Rancho Domingo, CA), then dried (Savant SpeedVac, Thermo Electron Corporation, Waltham, MA) and re‐dissolved in 100 *μ*L of assay buffer [1 nmol/L ^125^I‐Y‐Ugn in NaHCO_3_–free HBSS supplemented with 20 mmol/L sodium citrate and 0.75% bovine serum albumin (Sigma, St. Louis, Mo, USA)]. The pH was individually adjusted to 4.5 with NaOH or HCl using a miniaturized glass microelectrode (Microelectrodes Inc., Bedford, NH). Prepared samples were incubated in triplicate for 1 h at 25°C in polypropylene tubes with 20 *μ*L of T84 cell membranes (10–20 *μ*g protein). Samples were then diluted in 1 mL of ice‐cold rinse buffer, rapidly filtered through Whatman GF/C glass microfiber filters presoaked in distilled water, and quickly rinsed twice with 5 mL of ice‐cold rinse buffer (50 mmol/L Tris‐HCl at pH 7.0). Bound radioligand (retained by the filter) was quantified with a gamma counter (Packard Cobra, PerkinElmer Life and Analytical Sciences Inc., Waltham, MA). Ugn concentrations in sample solutions were calculated by interpolation in a standard curve obtained from a dilution series of synthetic rat Ugn incubated and analyzed in parallel with the unknowns. Non‐specific binding was determined by including 10^−5^ mol/L unlabeled Y‐Ugn in place of Ugn standards or unknowns.

### Data analysis

All data were plotted as mean ± SEM (or, in the case of *n* = 2, as mean ± range). Paired t‐tests were used for comparison of Ugn excretion, water consumption, and food consumption. GraphPad Prism 5.0 (LaJolla, CA) was used for analysis of binding data, with formulas derived from the Cheng‐Prusoff equation: cpm bound = NSB + [(*B*_max _− NSB)/1 + 10∧[log_10_(competitor concentration) − log_10_(IC_50_)]}], where NSB = nonspecific binding (in the presence of saturating competitor) and *B*_max_ = maximal binding (in the absence of competitor). For analysis of changes in gene expression by RT‐PCR, the 2^ΔΔCT^ calculation method was used (Livak and Schmittgen 2001). The final results represent the expression of Ugn/*β*‐actin transcript in tissue samples obtained during different dietary interventions, relative to the Ugn/*β*‐actin transcript expression during normal salt feeding prior to intervention.

## Results

### A novel assay for Ugn

Because GC‐C, the Ugn receptor (Schulz et al. 1990), is activated at the apical membranes of the cells that form the renal tubules (White et al. 1989; Hodson et al. 2006; Santos‐Neto et al. 2006), we can evaluate renal Ugn signaling (the presence of Ugn within the tubule lumen) by measuring the rate at which Ugn is excreted in urine (U_Ugn_V). However, the conventional Ugn bioassay, which measures levels of the peptide by quantifying its stimulatory effect on ligand‐activated cGMP synthesis (Carrithers et al. 1999, 2000a), is compromised by high levels of cGMP that are naturally present in urine. Therefore, we developed a competitive Ugn binding assay that is independent of cGMP. This method employs a synthetic rat Ugn analog radiolabeled with ^125^I, and plasma membranes derived from the T84 cell line, which abundantly expresses GC‐C. A further advantage of this new binding assay is that it is several orders of magnitude more sensitive than the bioassay.

**Figure 2:**
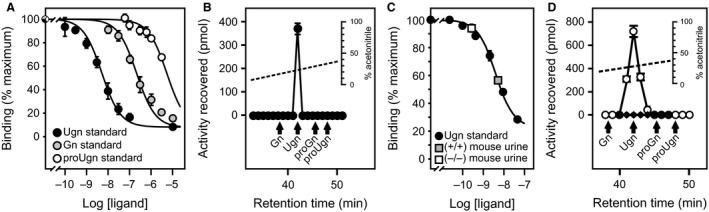
Validation of a novel Ugn binding assay. (A) Ability of synthetic proUgn, Ugn, and Gn to displace radioligand in the competitive binding assay. (B) The analyte responsible for the binding displacement activity of urine coelutes with authentic Ugn from a high‐resolution C‐18 HPLC column. A 200 *μ*L sample of urine (collected at 3 am on day 2 of a high‐salt diet) was loaded on the column; this is four times the volume that is loaded in a typical assay. Retention times of Gn, Ugn, proGn, and proUgn standards are indicated by the arrows. (C) Displacements of radioligand by urine samples from a Ugn knockout mouse and a wild‐type mouse are interpolated in a standard curve generated with a rat Ugn standard. (D) Endogenous Ugn is not detected in rat plasma (black symbols), whereas a strong peak is readily observed when 2 nmol exogenous Ugn are added to a plasma sample prior to chromatography (white symbols – retention times of Gn, Ugn, proGn, and proUgn standards are indicated by the arrows).

The properties of the new assay are presented in Table 3 and Figure 2. It can quantify Ugn in the picomolar range (Fig. 2A, black circles), allowing us to perform triplicate measurements of Ugn in rat urine samples as small as 20 *μ*L. Other peptides that cross‐react with GC‐C (Gn, proGn, and proUgn – see Fig. 2A) are not present in urine at detectable levels, even if the sample is taken under high salt conditions and overloaded by a factor of 4 (Fig. 2B). Assay specificity was further confirmed by comparing the displacement activities of urine obtained from wild‐type and Ugn knockout mice. Wild‐type mouse urine caused strong displacement of the labeled ligand (Fig. 2C, gray square), whereas the same volume of knockout mouse urine had no detectable activity (Fig. 2C, white square), confirming that urinary binding activity is dependent on the presence of an intact Ugn gene.

**Table 3:** Characteristics of the Ugn binding assay

Parameter	Value	*R* ^2^ (where applicable)	Number of determinations
Maximal binding (cpm)	1784.1 ± 87.4		(*n *=* *8)
Nonspecific binding (cpm)	134.0 ± 19.7		(*n *=* *8)
Assay reliability (SD of replicates as %mean)	3.3 ± 2.3%		(*n *=* *176)
K_d_ for ^125^I‐Y‐Ugn (mol/L)	1.5 × 10^−9 ^± 4.0 × 10^−10^	0.9659	(*n *=* *3)
K_i_ for rat Ugn (mol/L)	1.04 × 10^−9 ^± 0.032 × 10^−9^	0.9985	(*n *=* *8)
K_i_ for rat Gn (mol/L)	1.7 × 10^−7 ^± 4.4 × 10^−8^	0.9587	(*n *=* *3)
K_i_ for rat proUgn (mol/L)	8.9 × 10^−6 ^± 6.2 × 10^−7^	0.9677	(*n *=* *3)

Maximal binding was established with 1 × 10^−14^ moles ^125^I‐Y‐Ugn/µg membrane protein. Nonspecific binding was established using the same concentration of ^125^I‐Y‐Ugn in the presence of 1 × 10^−5^ mol/L unlabeled Ugn. Nonspecific binding was 7.5% of maximal binding. Assay reliability was determined by performing 176 independent triplicate measurements, and calculating the standard deviation of each determination as a percent of the mean value of the triplicates. Thus, the error of the assay is approximately 3%. Binding constants were determined for ^125^I‐Y‐Ugn and related peptides as described in the Methods.

### Ugn signaling activity in plasma

In past studies, using the previously established Ugn bioassay, we were unable to detect free Ugn in plasma (Moss et al. 2008; Qian et al. 2008). To see if plasma Ugn would be detectable with the more sensitive binding assay, we obtained a plasma sample at 3 am from an animal that had been consuming a high‐salt diet for 2 days (a time when urinary Ugn excretion is maximal; see below). We subjected this sample to C‐18 reverse‐phase HPLC, and measured the Ugn content of each fraction using the new assay (Fig. 2D, black symbols). No binding displacement activity was observed in any fraction collected at or near the elution time established for Ugn. To verify that Ugn would be detected if present, the analysis was also performed on plasma supplemented with 2 nmol of synthetic rat Ugn prior to the HPLC fractionation step. In this case, the exogenous Ugn produced a prominent peak of binding displacement activity (Fig. 2D, white symbols). Based on the recovery of the standard, the volume of plasma loaded onto the column, and the limit of detection of the assay, we estimate that the threshold concentration of Ugn in plasma that would have been detectable with this assay is ~60 fmol/mL. Given this limit of detection, circulating Ugn levels must therefore be less than 0.2% of circulating proUgn levels, which fluctuated between 2 and 5 pmol/mL in our studies (see Fig. 5, and discussion below). This result is consistent with previous studies demonstrating that proUgn is, by far, the dominant circulating form of the peptide (Moss et al. 2008; Qian et al. 2008).

### Ugn signaling activity in the kidney

**Figure 3:**
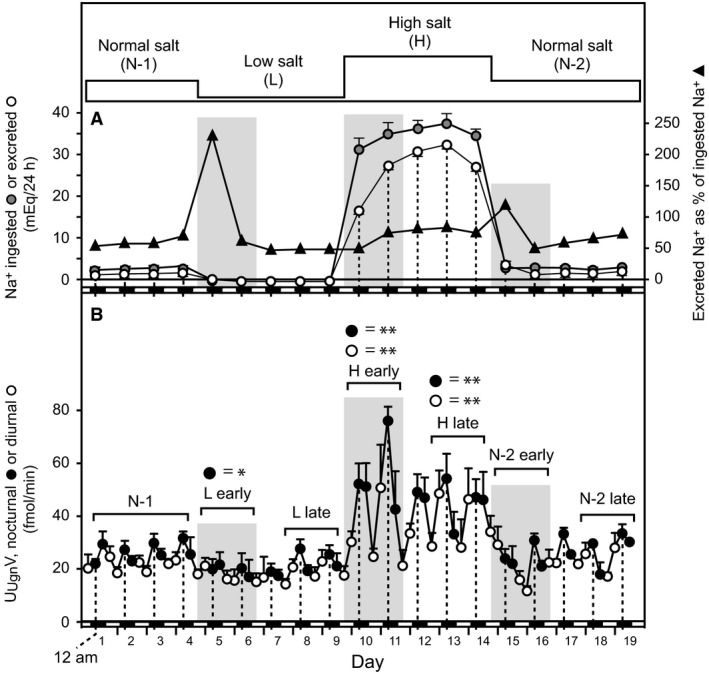
Urinary excretion of Ugn and Na^+^ as a function of diet. Longitudinal determination of Na^+^ consumption and urinary Na^+^ and Ugn excretion for rats fed sequentially on normal (N‐1), low (L), high (H), and normal (N‐2) Na^+^ diets. Vertical dotted lines correspond to 12 am (midnight), and the small black/white boxes along the horizontal axis indicate night/day status, respectively. (A) In the upper panel, the gray symbols indicate daily Na^+^ ingestion in mEq/24 h, the white symbols indicate daily urinary Na^+^ excretion in mEq/24 h, and the black symbols show excretion as a percentage of ingestion (*n *= 5). (B) The lower panel shows urinary Ugn excretion rate in fmol/min, as determined for each 6‐h collection period (black and white symbols indicate samples that were obtained during the dark and light periods, respectively, *n *= 4 or 5).

To explore the effects of dietary manipulations on renal Ugn signaling, we used the new binding assay to measure urinary Ugn excretion (U_Ugn_V) in animals that were fed chow containing normal levels of Na^+^ (NS‐1), then shifted to low‐Na^+^ chow (LS), followed by high‐Na^+^ chow (HS), and finally returned to normal Na^+^ chow (NS‐2). Throughout the study, Na^+^ consumption tracked the Na^+^ content of the food (Fig. 3A, gray symbols). Urinary Na^+^ excretion (U_Na_V) also tracked the dietary changes (Fig. 3A, white symbols), but as is typically observed in such studies (Rose 1994), a lag was observed relative to consumption, as it took ~2 days for animals to reestablish a stable ratio of excretion to ingestion after each dietary manipulation (Fig. 3A, black symbols; the gray shading highlights periods of transition from one equilibrium state to another). Note that urinary excretion was less than 100% of ingestion, even during the initial phase of the study when animals had not yet experienced any dietary salt challenges. This is because of Na^+^ excreted by nonrenal mechanisms, and, also, because calculated ingestion is slightly inflated due to the loss of small amounts of food by spillage. However, because we do not expect these factors to vary over time, we do not believe that they affect our estimate of the rate at which equilibrium is established.

U_Ugn_V showed a circadian rhythm throughout the study, with peak excretory rates occurring during the dark period, when the animals were awake, physically active, and consuming food (Fig. 3B). During the NS‐1 period, mean nocturnal and diurnal values for U_Ugn_V were 28.8 ± 1.2 and 23.0 ± 1.1 fmol/min, respectively (*P* << 0.001, nocturnal NS‐1 vs. diurnal NS‐1). Upon shifting to LS, nocturnal U_Ugn_V significantly decreased by 18% to 23.7 ± 0.4 (*P* = 0.002 vs. NS‐1) and diurnal U_Ugn_V decreased marginally to 20.6 ± 0.3 fmol/min (*P* > 0.05 vs. NS‐1). Because the decrease in nocturnal excretion was proportionally greater than the decrease in diurnal excretion, the circadian rhythmicity became less evident, and the nocturnal and diurnal values no longer differed significantly from each other (*P* > 0.05, nocturnal LS vs. diurnal LS). Upon shifting to HS, however, both nocturnal and diurnal U_Ugn_V significantly increased (by 97% to 56.8 ± 2.8 fmol/min for nocturnal HS and by 65% to 37.9 ± 3.3 fmol/min for diurnal HS, *P* << 0.001 for both vs. NS‐1), and the circadian rhythm re‐emerged robustly (*P* << 0.001, nocturnal HS vs. diurnal HS).

Closer inspection of Figure 3B reveals that U_Ugn_V was not uniform over the duration of each of the dietary manipulations. For example, as mentioned above, the circadian rhythm of U_Ugn_V was initially suppressed after the shift to low‐Na^+^ chow (days 5–7), primarily due to suppression of the nocturnal peak (*P* < 0.05, LS vs. NS‐1). However, on days 8–9, the nocturnal U_Ugn_V peak reappeared, and these values could no longer be distinguished from control nocturnal excretion (*P* > 0.05). Interestingly, the early “onset” phase of the U_Ugn_V response (suppressed nocturnal Ugn signaling) overlapped with the transition period when Na^+^ consumption and excretion were out of balance (gray shaded area at days 5–7 in Fig. 3), whereas the later “adapted” phase (the return toward control levels of nocturnal Ugn excretion) emerged as Na^+^ equilibrium was re‐established (days 8–9).

Similarly, when animals were shifted to high Na^+^ chow, nocturnal U_Ugn_V increased rapidly (days 10–11), but then dropped and stabilized at an intermediate level on days 12–14 (*P* < 0.05 for nocturnal U_Ugn_V on days 12–14 vs. day 11). Diurnal HS U_Ugn_V also increased, but plateaued after the first day and did not display the transient change observed for nocturnal values after each diet alteration (*P* > 0.05 for diurnal U_Ugn_V on days 12–14 vs. day 11). As with the shift from normal to low salt, the early onset phase (transiently enhanced nocturnal Ugn excretion) coincided with the transitional period when the new rate of Na^+^ excretion was being established (gray shaded area at days 10–11 in Fig. 3), while the later adapted phase (the return toward control nocturnal Ugn excretion) emerged once the new, steady‐state level of Na^+^ excretion had been achieved (days 12–14).

When the animals were returned to a normal salt diet at the end of the experiment (days 15–19), the effects of the high‐salt diet on U_Ugn_V reversed. As with the earlier dietary manipulations, it appears that nocturnal excretion of Ugn may have been transiently suppressed during the transition period (gray shaded area at days 15–16 in Fig. 3), though this could not be confirmed statistically due to the steep overall rate at which excretion was declining during this period. The decline was then followed by a return to the original circadian cycling of nocturnal and diurnal excretion as the animals once again became adapted to the normal salt diet.

### Relationship between U_Na_V and U_Ugn_V

**Figure 4:**
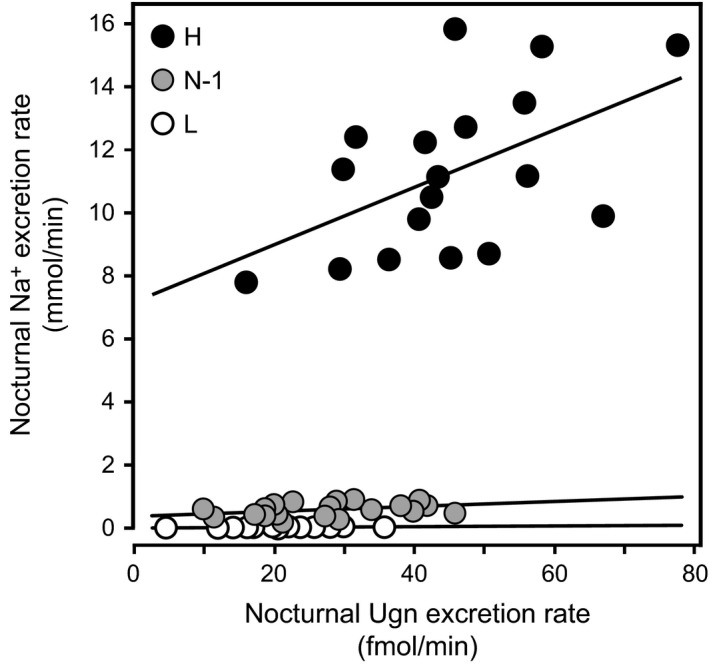
Urinary Na^+^ excretion as a function of urinary Ugn excretion under different dietary conditions. Each data point represents urinary salt excretion plotted as a function of contemporaneous urinary Ugn excretion for an individual animal at a specific time point. Data were obtained from nocturnal urine samples collected during different salt feeding phases (see text and Fig. 3; N‐1 = initial period of normal salt consumption, L = low salt consumption, H = high salt consumption). Lines were fit by linear regression. For high salt, *y *= 0.0799x + 6.2673, *R*² = 0.27; for normal salt, *y *= 0.0068x + 0.334, *R*² = 0.17; for low salt, *y *= 0.0009x + 0.02, *R*² = 0.39.

Figure 4 plots urinary Na^+^ excretion (U_Na_V) against urinary Ugn excretion (U_Ugn_V) for individual animals that were included in the study reported in Figure 3. The graph shows results obtained during the animals’ period of peak nocturnal activity (midnight – 6 am; data are reported only for those periods of the study in which animals had adapted to their diet, and the ratio of Na^+^ ingestion to Na^+^ excretion had reached a stable value [nonshaded areas in Fig. 3]. For rats on low‐salt diet, nocturnal Ugn excretion varied over a significant range from animal to animal, and was relatively weakly correlated with observed rates of Na^+^ excretion, as evidenced by the shallow slope of the linear regression curve (white symbols, slope = 0.0009, *P* < 0.006 that the slope is non‐zero, *R*² = 0.39). Ugn excretion fell within a slightly higher range of values when salt intake was normal, and under these dietary conditions, correlated more robustly with the slightly elevated rates of Na^+^ excretion (gray symbols, slope = 0.007, *P* < 0.04 that the slope is non‐zero, *R*² = 0.17). A further rightward shift in Ugn excretion and a large increase in Na^+^ excretion were observed when dietary salt intake was elevated above normal, and, here, the excretion rate of Ugn showed the strongest correlation with the excretion rate of Na^+^ (black symbols, slope = 0.08, *P* < 0.03 that the slope is non‐zero, *R*² = 0.27).

### Plasma proUgn measurements

The data in Figure 3 indicate that U_Ugn_V (and, thus, Ugn signaling within the kidney) is regulated by dietary salt. The entero‐renal signaling hypothesis described in the Introduction postulates that this excreted Ugn will have been delivered to the kidney as circulating proUgn that originated from the intestine (Fan et al. 1996; Moss et al. 2008; Qian et al. 2008). To test this, we measured the effects of various experimental manipulations on plasma levels of proUgn, using a highly specific, well‐validated western blot‐based immunoassay (see Methods, Figure 1, and published descriptions (Moss et al. 2008, 2010; Qian et al. 2008)). Our initial approach involved vascular ligation studies to establish the tissue source of plasma proUgn.

### Effects of tissue ligations on plasma proUgn levels

**Figure 5:**
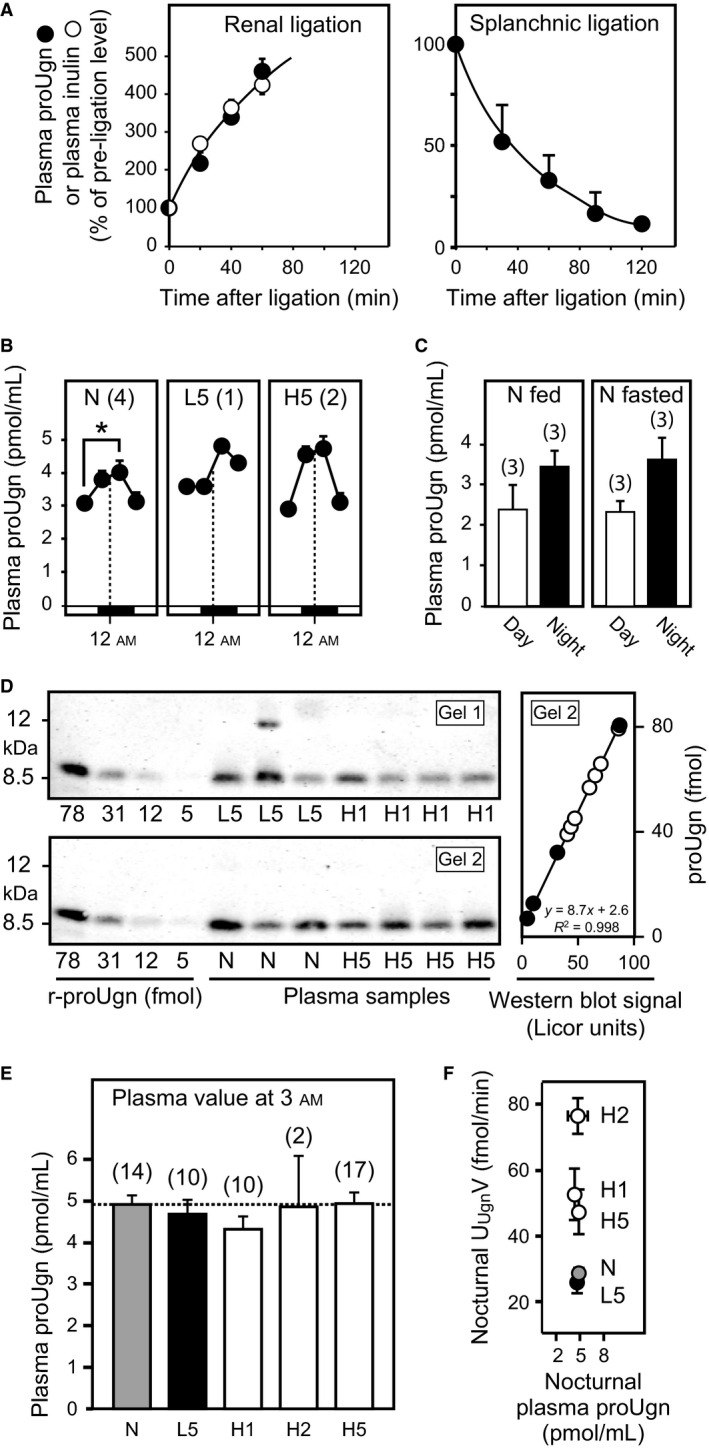
Western blot analysis of plasma proUgn. The quantitative western blot assay is described in the Methods and in Figure 1. The number of replicates (*n*) is given in parentheses for each measurement. (A) Time course of plasma levels of proUgn (black circles) and inulin (white circles) after ligation of splanchnic or renal arteries, as indicated. (B) Circadian fluctuations in circulating plasma levels of proUgn are illustrated for a subset of the animals included in the diet study. Data were acquired from rats fed normal chow (*N*), after 5 days on low‐salt chow (L5), and after 5 days on high‐salt chow (H5). Plasma samples (100 *μ*L) were taken every 6 h via an indwelling carotid cannula, and assayed for proUgn content using the quantitative western blot assay. Data points are plotted against the time of sample acquisition. The dashed line indicates 12 am (midnight). Asterisk indicates *P *< 0.05. (C) Diurnal (white bars) and nocturnal (black bars) plasma levels of proUgn in animals fed ad libitum (left panel) or animals fasted (right panel) for 12 h (diurnal fasting values) or 24 h (nocturnal fasting values). (D) Representative western blots, showing a subset of the raw data used to generate the graphs in panels e and f. For quantitation, the infrared signal intensity of each sample was interpolated in a standard curve derived from known amounts of recombinant rat proUgn, as shown at the right for gel 2 (black symbols = standards, white symbols = plasma samples). Values from multiple gels could be combined to generate the final graphs in panels e and f, because every gel was loaded with an identical standard curve. (E) Circulating levels of proUgn at 3 am under defined dietary conditions. (F) Average rates of nocturnal urinary Ugn excretion (U_U__gn_V) plotted as a function of average nocturnal plasma proUgn concentration for animals consuming normal salt (NS), low salt for 5 days (LS5), or high salt for 1 (HS1), 2 (HS2) or 5 (HS5) days (*R*² = 0.009 for a linear fit to the data).

Previous surveys have identified only two organs (intestine and kidney) that contain levels of proUgn polypeptide sufficient to generate the relatively large circulating pool of proUgn (Moss et al. 2008; Qian et al. 2008). To determine the extent to which each of these organs contributes to the plasma pool, we ligated either the splanchnic or the renal blood supply, and measured the effects of each ligation on circulating proUgn levels. As shown in Figure 5A (right panel), plasma proUgn fell to barely detectable levels after splanchnic ligation, indicating that the intestine is by far the major (and possibly the exclusive) source of circulating propeptide. In contrast, plasma proUgn levels rose rapidly after ligation of the renal arteries (Fig. 5A, left panel, black symbols). For the renal ligations, we also infused each animal with inulin (a reference standard used to assess glomerular filtration rates), and measured plasma inulin at each time point (Fig. 5A, left panel, white symbols). On a percentage basis, the postligation rate of rise of plasma proUgn was nearly identical to that of inulin, a substance freely filtered and neither secreted into nor reabsorbed from the tubules. If the kidneys had been secreting proUgn into the plasma, then postligation changes in circulating proUgn levels would have lagged behind those of inulin, because the rate of proUgn delivery would have been decreased by the ligation, whereas inulin delivery was clamped at a constant rate by the infusion. Thus, with respect to plasma proUgn, the role of the kidney appears to be exclusively vascular clearance.

### Effects of dietary manipulations on plasma proUgn levels

Having identified the source of plasma proUgn, we then investigated temporal regulation of plasma proUgn levels. Figure 5B (left panel) shows measurements of proUgn in plasma sampled at regular intervals over a 24‐h cycle from rats maintained on a NS diet. Circulating levels ranged from 3 to 5 pmol/mL, in good agreement with previous results reported with this assay (Moss et al. 2008, 2010; Qian et al. 2008). In addition, nocturnal levels were consistently 30–40% higher than diurnal levels (*P* < 0.05), with fluctuations in amplitude comparable to those shown above for urinary Ugn in animals on the NS diet (Fig. 3B). The circadian rhythmicity observed for plasma proUgn levels also correlates well with the circadian oscillations reported for tissue levels of proUgn mRNA and proUgn polypeptide in the intestine (Scheving and Jin 1999), and is compatible with a recent study showing that plasma levels of proUgn rise transiently in response to food intake (Valentino et al. 2011), which in our experiments, occurred preferentially during the dark period (data not shown). However, plasma proUgn levels (diurnal and nocturnal) in animals maintained on standard chow were indistinguishable from those in animals that had been fasted for 12–24 h (Fig. 5C), indicating that there is either no acute effect of dietary intake on circulating propeptide levels, or that any such effect must be highly transient in nature (and therefore not detectable against the background levels observed during our six hour collection periods).

In evaluating whether chronic manipulation of dietary salt content would have any long‐term effect on plasma proUgn levels, we had initially hoped to obtain a complete set of longitudinal plasma propeptide measurements to compare with the longitudinal urinary Ugn measurements presented in Figure 3B. However, we were unable to maintain the patency of our arterial sampling ports for more than a few days. In addition, we became concerned that removal of four large (100 *μ*L) blood samples every day would have deleterious effects on the animals’ health over time, and might adversely affect the composition of their plasma proteome. Therefore, in a pilot study, we took a limited set of samples over a 24‐h period from a small number of individual animals maintained on each diet to investigate whether circadian rhythmicity was maintained for all treatment groups. We found that peak plasma proUgn values occurred at 3 am in each case [Fig. 5B compares the normal salt diet (N) to 5 days on low salt (L5) or 5 days on high salt (H5)].

Although the number of animals analyzed in Figure 5B was small, peak nocturnal plasma proUgn levels did not appear to be significantly influenced by dietary salt. To investigate this in a statistically reliable manner, we obtained single plasma samples from a larger number of individual animals at a specific time of day (3 am) under each dietary condition. This time point was selected on the basis of a previous kinetic study that revealed close temporal correlation between the intravascular infusion of a bolus dose of exogenous proUgn and the corresponding appearance of Ugn in the urine, with nearly all of the recovered Ugn appearing within 30–60 min of infusion (Moss et al. 2010). This indicates that any plasma peak of proUgn should occur approximately contemporaneously with the urinary peak of Ugn, if the former serves as the precursor for the latter. Accordingly, for this study, we sampled each animal at 3 am on either a normal salt diet (N), after 5 days of low‐salt diet (L5), or after 1, 2, or 5 days of high‐salt diet (H1, H2, and H5). Representative western blots from this study are shown in Figure 5D. After quantifying and pooling the entire data set, peak nocturnal levels of plasma proUgn were found to be strikingly independent of salt intake (Fig. 5E), suggesting strongly that the diet‐dependent fluctuations in urinary Ugn excretion observed in Figure 3B were not a consequence of diet‐dependent fluctuations in plasma proUgn. Indeed, when we plot nocturnal urinary Ugn excretion as a function of nocturnal plasma proUgn (Fig. 5F), no relationship can be observed between the two variables (*R* ^2 ^= 0.009). These results are consistent with more limited data from a previous study with mice, in which plasma proUgn levels also did not increase when the animals were subjected to a high‐salt diet for 2 or 4 days (Elitsur et al. 2006).

### Effects of dietary Na^+^ on steady‐state proUgn mRNA expression levels in intestine and kidney

**Figure 6:**
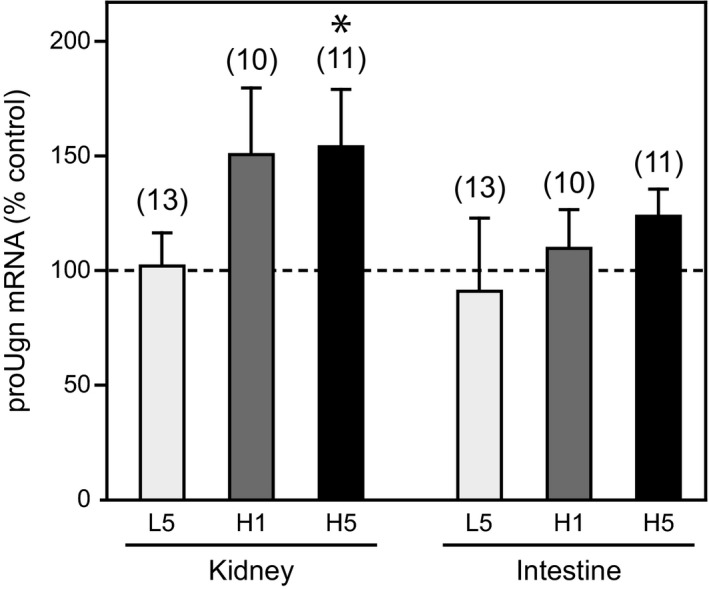
Ugn mRNA expression in the kidneys and small intestines of animals consuming normal, low, and high salt diets. All tissues for these mRNA measurements were collected from animals killed at 3 am (*n* is given in parentheses; asterisk indicates *P *< 0.05 vs. control). Expression of Ugn mRNA transcript is given as a percent of control (normal salt) levels in either kidney or small intestine when animals were fed a low‐salt diet for 5 days (L5), 24 h after switching to a high‐salt diet (H1), or 5 days after switching to a high‐salt diet (H5). The 2^ΔΔCt^ method was used to determine relative expression, as described in Methods.

The data presented above strongly suggest that the elevated levels of Ugn excreted in urine in response to increased consumption of dietary salt do not originate from the pool of proUgn circulating in plasma, and thus not from the pool of proUgn produced by the intestine. As mentioned above, the only tissue other than the intestine that contains a significant amount proUgn is the kidney (Qian et al. 2000, 2011; Moss et al. 2008). Therefore, as an independent way to assess diet‐responsive Ugn signaling activity in each of these tissues, we measured steady‐state tissue levels of proUgn mRNA in kidney, and compared it to those in the intestine. Renal proUgn mRNA content after 1 or 5 days on the HS diet was higher than in control animals (Fig. 6, left side). In contrast, enteric proUgn mRNA levels were not significantly affected by the HS diet (Fig. 6, right side). Interestingly, the LS diet had no demonstrable effect on Ugn mRNA in either kidney or intestine.

## Discussion

### Does Ugn mediate an entero‐renal natriuretic reflex that responds to salt ingestion?

The hypothesis that Ugn serves as the agent of a natriuretic endocrine axis that links the intestine to the kidney has been widely promoted in the Ugn literature (Forte et al. 1996, 2000; Krause et al. 1997; Potthast et al. 2001; Forte 2003; Elitsur et al. 2006; Qian et al. 2008; Lima and Fonteles 2014). Recent speculation has focused on proUgn, rather than Ugn, as the endocrine mediator of this putative signaling axis, because (1) proUgn is by far the most abundant molecular species stored in tissues and circulating in plasma (Moss et al. 2008), and (2) when isotopically labeled recombinant proUgn is infused intravascularly, it is readily filtered from plasma by the kidney, and converted within the kidney to Ugn (Qian et al. 2008). However, one critical prerequisite for accepting this entero‐renal signaling hypothesis – direct confirmation that oral salt ingestion triggers enteric proUgn secretion – remains controversial.

Our current studies demonstrate that salt ingestion does, indeed, activate Ugn‐based signaling. However, our data strongly suggest that this occurs within the kidney rather than within the intestine. A number of observations, taken together, support this assertion. First, urinary Ugn excretion was modestly suppressed in salt‐restricted animals, and greatly enhanced in salt‐challenged animals, demonstrating that salt intake does regulate Ugn levels within the tubules of the nephron. However, plasma proUgn levels did not change when dietary salt levels were manipulated (Fig. 5B,D,E,F), and were not affected by a 12–24 h fast (Fig. 5C). Indeed, plasma proUgn levels were essentially identical under every dietary condition that we tested (see Fig. 5F), while U_Ugn_V varied over a fourfold range in response to the same diets (Fig. 3B). This argues that urinary Ugn is unlikely to be derived from plasma proUgn, because changes in U_Ugn_V over this range without corresponding changes in plasma proUgn levels could only occur if GFR varied proportionally over the same fourfold range in response to each of the dietary manipulations – but numerous published studies have shown that GFR is unresponsive, or only marginally responsive, to long term changes in dietary salt load (Koepke and Dibona 1985; Koepke et al. 1988; Shirley and Skinner 1994; Isaksson et al. 2014). Furthermore, elevated Ugn mRNA levels in the kidneys of animals consuming a high salt load (Fig. 6) could plausibly drive enhanced renal production of the proUgn needed to support the increased rate of urinary Ugn excretion that is observed under these dietary conditions (Fig. 3B), while enteric levels of Ugn mRNA were unchanged after 5 days on the high‐salt diet (Fig. 6). It might be argued that the salt‐consuming animals in our study did display a slight upward trend in intestinal Ugn mRNA expression. However, corroborating evidence that enteric Ugn expression is insensitive to dietary salt is provided by an independent study that showed little or no difference in Ugn mRNA levels (<3.5% increase) in any gastrointestinal tissue (duodenum, jejunum, ileum, and colon) relative to control after shifting rats to a high‐salt diet for 4 days (Carrithers et al. 2002).

Thus, in rats, plasma proUgn does not fulfill the basic requirements expected of a circulating natriuretic agent with an entero‐renal endocrine function. However, two caveats should be mentioned. First, we cannot fully rule out the possibility that Ugn, itself, may serve as the circulating mediator of such an axis. We were unable to directly test this hypothesis in our current studies, as plasma Ugn levels fell below the limit of detection for the most sensitive assay available to us (Fig. 2D). However, we do not favor this idea for two compelling reasons. First, it seems unlikely that a diet‐dependent change in the rate of secretion of Ugn from the intestine could occur without a parallel change in the rate of synthesis and secretion of proUgn (which we do not observe), since the peptide and its propeptide precursor would almost certainly be packaged in the same secretory granules, and therefore cosecreted. Second, although our binding assay was not sensitive enough to detect Ugn in plasma, a previous study using a much more sensitive RIA procedure has reported no difference in circulating levels of Ugn between rats maintained for 7 days on a low‐salt diet (0.08% NaCl) and rats maintained for 7 days on a high‐salt diet (4% NaCl) (Fukae et al. 2002). Although the 7‐day time point reported in this published study does not exactly match the time parameters of our current work, the published data do lend strong support to the idea that plasma Ugn levels do not fluctuate in response to long‐term changes in dietary salt intake.

A second caveat concerns the possibility that the postsecretory processing mechanism(s) that convert plasma proUgn to Ugn may themselves be regulated by diet. Thus, a constant pool of plasma proUgn could be converted to a varying urinary pool of urinary Ugn if, for example, a high‐salt diet were to upregulate a renal enzyme that converts filtered plasma proUgn to Ugn and/or downregulates an enzyme that degrades Ugn after its production from plasma proUgn. Indeed, a recently described processing mechanism that converts circulating proUgn to active form within the hypothalamus provides a potential source of plasma Ugn (Valentino et al. 2011), though the biochemical tracer studies cited above have shown directly that this mechanism does not return significant levels of Ugn from the CNS (or any other tissue) to the general circulation (Qian et al. 2008). Furthermore, any proteolytic processing that is postulated to operate outside of the intestine could not, by definition, serve as a route for communication between the intestine and the kidney, because up‐ or down‐modulation of the processing enzyme (the critical step in regulating Ugn‐based signaling activity) would necessarily occur outside of the intestine, and thus would be under the control of some other tissue.

### Salt sensitivity of the Ugn signaling system

One important observation emerging from our study is that intratubular Ugn signaling appears to play a more prominent role in the context of increased salt consumption than it does during periods of normal or restricted consumption. As shown in Figure 4, the slope of the curve that compares urinary Ugn excretion rates to contemporaneous urinary Na^+^ excretion rates is steepest under conditions of high salt consumption (black symbols), whereas the relationship is less pronounced when salt intake is normal or suppressed (gray and white symbols, respectively). Similarly, as shown in Figure 3B, there is only a small decrease (−18%) in nocturnal urinary Ugn excretion on the low‐salt diet relative to the normal salt diet, whereas a much larger increase (+97%) is observed on the high‐salt diet. The relatively small effect during a low‐salt diet is accompanied by negligible effects on renal expression of proUgn at the mRNA level, in contrast to the prominent effects of the high‐salt diet on this parameter (Fig. 6). The most straightforward interpretation of these observations is that, at normal and subnormal levels of intake, Na^+^ excretion is regulated primarily by Ugn‐independent mechanisms, such as the actions of renin–angiotensin–aldosterone system (RAAS), and Ugn does not play a prominent role at these times. However, when dietary salt intake is elevated, the RAAS is strongly suppressed (Gibbons et al. 1984; Skott and Briggs 1987; Damkjaer et al. 2013), and renal synthesis of proUgn is enhanced (Fig. 6), leading to elevated delivery of Ugn to the lumen of the nephron, and allowing a strong correlation between urinary Ugn excretion and urinary sodium excretion to emerge.

### The characteristics of the Ugn response to ingested salt are consistent with the phenotype of the Ugn knockout mouse

When salt intake was manipulated in our study, rats exhibited two distinct phases of altered nocturnal urinary Ugn excretion: an early “onset” phase that lasted for approximately 2–3 days, while the animals were equilibrating Na^+^ output with the new level of intake, and a later “adapted” phase that emerged as the new equilibrium was established. The latter phase lasted for as long as the diet remained constant. This correlates well with a prominent aspect of the phenotype of the Ugn knockout mouse, as Na^+^ excretion in these animals lags behind that of the wild‐type animals by roughly 2 days after a shift to a high‐salt diet (Lorenz et al. 2003). Our data suggest that the natriuretic response of the mutant animals may be delayed because they lack the rapid natriuretic stimulus provided by the early phase of Ugn production, while their eventual ability to achieve Na^+^ balance reflects the actions of other homeostatic mechanisms that are independent of the Ugn system.

Furthermore, the chronic hypertension displayed by Ugn knockout mouse (Lorenz et al. 2003), could result from the absence of a chronic natriuretic action of Ugn. In this regard, it is clear from Figure 3B that animals maintain a measurable level of urinary Ugn production at all times, including during periods of severe salt‐restriction. It therefore seems likely that eliminating all Ugn‐mediated salt excretion, as would be the case in the Ugn knockout mouse, would require a compensatory response to substitute for the absence of Ugn. This could involve an increase in blood pressure to a new set‐point where the resultant pressure natriuresis would increase salt excretion to match intake. Thus, our data provide mechanistic insight into how the lack of renally produced Ugn might generate both the hypertension and the delay in salt excretion after a salt challenge that are observed in the Ugn knockout mouse.

It has also been reported that the elevated blood pressure of the Ugn knockout mouse is not affected by the manipulation of dietary salt (Lorenz et al. 2003). Although our data do not straightforwardly predict this aspect of the mutant phenotype, it is worth considering that U_Ugn_V displayed two distinct phases in our study: a nocturnal phase that was conspicuously affected by dietary salt, and a diurnal phase that was relatively unaffected (Fig. 3b). Perhaps loss of the latter has more impact on the overall phenotype of the mutant animals than does loss of the former.

### Anatomical considerations

In the rat, renal proUgn has been localized to a subset of epithelial cells that is restricted to the distal convoluted tubules and cortical collecting ducts (Qian et al. 2011). Although the bulk of the Na^+^ that enters the nephron is typically reabsorbed within the proximal tubules, enhanced dietary Na^+^ intake will shift the salt profile within the tubule so that the rate of salt delivery to cells of the distal nephron will increase. Studies in Sprague‐Dawley rats have shown that a high‐salt diet can blunt the ability of the kidney to auto‐regulate renal blood flow (Fellner et al. 2014), which would lead to transient increases in distal nephron salt delivery as blood pressure fluctuates upwards. In this context, it is interesting to note that salt stimuli increase the abundance of Ugn mRNA in a Ugn‐expressing epithelial cell line (Steinbrecher et al. 2002). Thus, a diet‐dependent shift in intratubular salt delivery may serve as a mechanism that underlies the changes in renal Ugn mRNA expression observed in our study (Fig. 6). As an additional consideration, the distal nephron contains functionally significant levels of prostasin, a luminally oriented protease that has the appropriate specificity to generate bioactive Ugn from proUgn (Yu et al. 1995; Vallet et al. 1997; Kitamura and Tomita 2010). Any Ugn produced from proUgn at these strategic intratubular sites would have direct access to distal portions of the nephron, where it could act in concert with other key sodium‐ and volume‐regulatory hormones, such as atrial natriuretic peptide, aldosterone, and antidiuretic hormone. Thus, both a potentially appropriate stimulus (increased luminal salt delivery) and a potential processing mechanism (luminally oriented prostasin) are available in the distal nephron where renal proUgn is expressed.

In rats, the Ugn receptor (GC‐C) is expressed to varying degrees along the length of the nephron, but the highest levels are found in the distal collecting tubules (Carrithers et al. 2000), consistent with the distal actions of Ugn described above. However, renal responses to Ugn (and normal blood pressure) are preserved in the GC‐C knockout mouse, arguing that an alternate, as‐yet unidentified Ugn receptor must exist (Carrithers et al. 1999, 2004). This alternate receptor appears by far to be the dominant Ugn receptor in the rat kidney (Qian et al. 2011), and its intrarenal expression pattern is not yet known.

### Perspectives

Results presented in this communication support the conclusion that an intrarenal pool of proUgn is converted to an intratubular pool of Ugn in a diet‐dependent manner. In conjunction with previous studies, this observation critically strengthens the contention that Ugn is an authentic endocrine agent as follows: (1) Ablation of the Ugn gene leads to hypertension (Lorenz et al. 2003; Elitsur et al. 2006), an appropriate phenotype for an animal that lacks an endocrine mechanism involved in electrolyte homeostasis. (2) Ugn itself evokes a natriuretic response when delivered to the lumen of the nephron by glomerular filtration (Greenberg et al. 1997; Carrithers et al. 1999; Elitsur et al. 2006; Moss et al. 2009, 2010). Thus, exogenously delivered peptide mimics the actions of the proposed endogenous signaling pathway. (3) The Ugn precursor (proUgn) is synthesized by specific cells that are found in the distal convoluted tubules of the kidney (Qian et al. 2011). These Ugn‐expressing cells are therefore localized to a functionally appropriate anatomical location, consistent with their hypothesized regulatory role. (4) Pertinent dietary manipulations alter the renal synthesis of proUgn mRNA, the conversion of proUgn to Ugn, and the secretion of Ugn into the tubule lumen (our new data, presented in Figs. 3 and 6), providing direct evidence that the activity of the Ugn signaling pathway is modulated by dietary stimuli. Taken together, the data strongly support the hypothesis that renal Ugn signaling is one component of the multi‐hormonal regulatory system that balances Na^+^ excretion with Na^+^ intake. The data do not support the hypothesis that enterically produced proUgn participates in this regulatory system, though we cannot completely rule out this possibility. The intriguing question of why such high levels of proUgn are secreted into the circulation by the intestine remains to be answered. Perhaps, the function(s) of this large plasma pool of propeptide are to provide circadian control over hunger by acting on the brain (Valentino et al. 2011), to drive basal (non diet‐dependent) circadian excretion of salt by the kidney, and/or to play a role in overall energy balance, as suggested by a recent study showing that intestinal proUgn levels are elevated by a high fat diet, depressed in leptin‐deficient animals, and elevated by treatment with exogenous leptin (Folgueira et al. 2016).

## Conflict of Interest

The authors have nothing to disclose.
